# Deficient prefrontal-amygdalar connectivity underlies inefficient face processing in adolescent major depressive disorder

**DOI:** 10.1038/s41398-022-01955-5

**Published:** 2022-05-10

**Authors:** David Willinger, Iliana I. Karipidis, Isabelle Häberling, Gregor Berger, Susanne Walitza, Silvia Brem

**Affiliations:** 1 Department of Child and Adolescent Psychiatry and Psychotherapy, University Hospital of Psychiatry Zurich, University of Zurich, Zurich, Switzerland; 2 Neuroscience Center Zurich, University of Zurich and ETH Zurich, Zurich, Switzerland; 3 Center for Interdisciplinary Brain Sciences Research, Stanford University School of Medicine, Stanford, CA USA

**Keywords:** Depression, Diagnostic markers, Human behaviour

## Abstract

Adolescence represents a critical developmental period where the prevalence of major depressive disorder (MDD) increases. Aberrant emotion processing is a core feature of adolescent MDD that has been associated with functional alterations within the prefrontal-amygdala circuitry. In this study, we tested cognitive and neural mechanisms of emotional face processing in adolescents with MDD utilizing a combination of computational modeling and neuroimaging. Thirty adolescents with MDD (age: M = 16.1 SD = 1.4, 20 females) and 33 healthy controls (age: M = 16.2 SD = 1.9, 20 females) performed a dynamic face- and shape-matching task. A linear ballistic accumulator model was fit to the behavioral data to study differences in evidence accumulation. We used dynamic causal modeling (DCM) to study effective connectivity in the prefrontal-amygdala network to reveal the neural underpinnings of cognitive impairments while performing the task. Face processing efficiency was reduced in the MDD group and most pronounced for ambiguous faces with neutral emotional expressions. Critically, this reduction was related to increased deactivation of the subgenual anterior cingulate (sgACC). Connectivity analysis showed that MDD exhibited altered functional coupling in a distributed network spanning the fusiform face area–lateral prefrontal cortex–sgACC and the sgACC–amygdala pathway. Our results suggest that MDD is related to impairments of processing nuanced facial expressions. Distributed dysfunctional coupling in the face processing network might result in inefficient evidence sampling and inappropriate emotional responses contributing to depressive symptomatology. Our study provides novel insights in the characterization of brain function in adolescents with MDD that strongly emphasize the critical role of aberrant prefrontal-amygdala interactions during emotional face processing.

## Introduction

Major depressive disorder (MDD) is a severe, highly disabling mental disorder with drastic impairments in psychosocial functioning causing high social and economic costs [1]. First onset often coincides with a critical, stress-sensitive period of brain development during adolescence [2, 3] and at 17–18 years its lifetime prevalence is up to 13.5% [4, 5]. MDD has one of the highest disease burden in young people [6] and a dramatically increased suicidality [7]. A better understanding of the underlying neurobiology is essential for the improvement of treatment and prevention of MDD in youth.

Ample evidence suggests that MDD is associated with a cognitive negative bias associated with maladaptive evaluation and behavior [8–11]. Especially in youth at familial risk, such bias is most likely an important vulnerability factor that facilitates the occurrence of MDD [12]. A growing body of literature has linked this cognitive bias during emotion processing to a dysregulation of the prefrontal-amygdala network [13, 14]. During adolescence, the prefrontal-amygdala network has a stress-sensitive developmental trajectory that is not only associated with increased risk for depressive symptoms but also with alterations in emotion recognition and regulation (see Tottenham et al. [15] for review). Early studies showed that adolescents at risk for [16, 17] and with MDD [18–23] exhibit increased amygdala reactivity during emotional face processing. Moreover, adolescent MDD has been associated with aberrant activity in the subgenual anterior cingulate cortex (sgACC) [24–26] and the lateral prefrontal cortex (LPFC) [27] consistent across a variety of emotion processing tasks. Additionally, similar to adult MDD [28–32], emerging evidence indicates that the functional coupling within the prefrontal-amygdala network is disrupted in adolescents with MDD [25, 33, 34] and depressive symptoms [35]. In particular, findings of Ho et al. [25] and Musgrove et al. [33] suggest connectivity alterations between sgACC and amygdala and decreased connectivity between sgACC and fusiform face area (FFA) in youth with MDD during emotional face processing. In a recent longitudinal study, Jamieson et al. [34] found that prefrontal-amygdala interactions not only characterize adolescent depression but also predict treatment response using an implicit emotional processing task with sad and fearful faces. Although the emotional paradigms in these studies slightly varied, these results indicate a dysbalance that may underpin alterations in emotion processing. Nevertheless, it remains unclear whether connectivity alterations in youth MDD generalize to neutral and positive valence. Characterizing the function of valence in prefrontal-amygdala interactions in healthy and affected adolescents, as we have done previously in healthy adults [36], will help to further improve our understanding of the neural correlates of MDD.

In pursuit of an integrated and thorough understanding of cognitive and neural mechanisms, the present study aimed at harnessing latest methodological advances using a combination of a choice response time (RT) model and dynamic causal modeling (DCM) to characterize emotion processing in adolescent MDD. Choice RT models use both response time and accuracy to divide the behavioral data of simple decision processes into individual components (e.g. response caution, processing efficiency). This allows for relating components of the modeled decision process to functional brain networks, enabling an abstract, mechanistic interpretation not only on behavioral but also on a neural level. Recent work has shown that employing computational choice RT models can reveal latent cognitive mechanisms in MDD [37], improving the sensitivity of analysis, and can uncover associations with brain function [38]. Thus, it provides a unique approach to comprehensively investigate the functional integration of emotional information in the brain.

We aimed to study the functional architecture of the prefrontal-amygdala circuitry in adolescent MDD using a dynamic emotional face matching task. An effective connectivity analysis was performed to investigate the neural dynamics associated with information processing difficulties in patients with depression. We hypothesized that adolescents with MDD show aberrant emotion processing, reflected by differences in evidence accumulation [38]. In addition, we tested whether this deficiency in perceiving emotions is related to altered connectivity within the prefrontal-amygdala network. We expected to find (a) disrupted connectivity between cortical regions sgACC, LPFC and fusiform face area (FFA) [25, 33], and (b) reduced top-down influence of prefrontal regions to the amygdala in MDD [32]. Finally, in concordance with previous work in adults [36], we hypothesized that negative and positive valence engage the prefrontal-amygdala network differentially.

## Methods and materials

### Participants

Thirty MDD patients and 33 healthy individuals matched for age, IQ, sex, and handedness participated in this study (Table 1). We conducted a semistructured clinical interview (Schedule for Affective Disorders and Schizophrenia for School-Age Children–Present and Lifetime Version, Kiddie-SADS [39], or Mini-International Neuropsychiatric Interview for Children and Adolescents, MINI-KID [40]) with all participants. For control subjects, exclusion criteria included any current psychiatric disorder or other major medical illnesses, drug abuse, any MRI contraindication, pregnancy, and a history of brain injury. Patients received individual psychotherapeutic support as needed during the time of the study. All participants gave their written informed consent and were financially reimbursed at the end of the study. The authors assert that all procedures contributing to this work comply with the ethical standards of the relevant national and institutional committees on human experimentation and with the Helsinki Declaration of 1975, as revised in 2008.

**Table 1:** Clinical and demographic characteristics of study participants.

	Controls	MDD	Test statistic	*p* value^a^
Age (years), range (min-max)	16.2 (1.9), 11.2–18.8	16.1 (1.4), 12.8–18.7	U = 553.5	0.425
Sex (males), No. (%)	10 (30%)	10 (33%)	χ^2^(1) = 0.07	0.796
Handedness (right), No. (%)	32 (97%)	28 (93%)	χ^2^(1) = 0.46	0.500
In-scanner movement (FD, mm)	0.16 (0.06)	0.17 (0.06)	t(61) = 0.69	0.492
CD-RISC	72.9 (10.1)	38.6 (15.6)	t(58) = 10.16	<0.001
CDI	8.4 (6.6)	29.6 (9.3)	U = 38.0	<0.001
Anhedonia	2.3 (2.2)	10.5 (2.8)	U = 13.5	<0.001
Negative mood	2.2 (2.0)	6.4 (2.4)	U = 88.0	<0.001
Negative self-esteem	1.0 (1.2)	5.0 (1.7)	U = 42.0	<0.001
Ineffectiveness	1.2 (1.2)	5.0 (1.9)	U = 54.5	<0.001
Interpersonal problems	1.1 (1.2)	3.7 (1.5)	U = 74.5	<0.001
Stomach	0.6 (0.6)	1.1 (0.8)	U = 301.5	0.018
RIAS IQ	104.5 (6.9)	108.0 (8.7)	t(60) = −1.75	0.079
PSS	22.4 (6.6)	28.8 (7.7)	t(57) = −3.44	0.001
SDQ	8.8 (5.3)	16.3 (5.6)	t(56) = −5.26	<0.001
WISC-IV Digitspan (forward)	8.9 (2.1)	8.8 (2.0)	t(60) = 0.32	0.747
WISC-IV Digitspan (backward)	8.6 (1.6)	9.4 (2.0)	t(60) = −1.70	0.094
WISC-IV Mosaic	57.0 (5.7)	59.0 (6.2)	t(56) = −1.27	0.208
Current Medication, No. (%)				
No medication	33 (100%)	10 (33%)	NA	NA
SSRI	0	18 (60%)	NA	NA
Dual-action antidepressant^b^	0	2 (7%)	NA	NA
NERI	0	2 (7%)	NA	NA
Antipsychotic^c^	0	2 (7%)	NA	NA
Methylphenidate	0	2 (7%)	NA	NA

Data are presented as mean (SD) if not indicated otherwise.

*CD-RISC* Connor-Davidson Resilience Scale, *CDI* Children Depression Inventory, *FD* framewise displacement, *NERI* Norepinephrine reuptake inhibitor, *PSS* Perceived Stress Scale, *RIAS* Reynolds Intellectual Assessment Scales, *SDQ* Strength and Difficulty Questionnaire for Children, *SSRI* Selective serotonin reuptake inhibitor, *WISC* Wechsler Intelligence Scale for Children.

^a^Uncorrected *p* values for between-group comparisons; significance threshold *p* < 0.05.

^b^Serotonin-noradrenalin reuptake inhibitor.

^c^Used for behavioral control.

### Experimental task

In this study, healthy controls and participants with MDD performed a dynamic face- and shape-matching task, that has higher ecological validity and has been shown to yield stronger prefrontal-amygdala network activation [41, 42] than the often-validated static task [43] (Fig. S1). The probes at the bottom were matched to the dynamic stimulus slowly changing from a neutral expression to the target emotion at the top. Subjects were presented with 4 blocks of 5 trials each for each condition (positive, negative, neutral, shapes) in randomized order, resulting in 80 trials in total. To create the neutral condition, we presented faces that were rated either as neutral (valence rating from Langner et al. [44], scale: 1 (negative) – 5 (positive); M = 2.95, SD = 0.02) or contemptuous (M = 2.79, SD = 0.05), to ensure a set of nuanced dynamic neutral expressions. For the positive condition, we used happy faces (M = 4.60, SD = 0.15) and the most positively rated surprised faces (M = 3.04, SD = 0.06). Sad (M = 1.80, SD = 0.05) and disgusted (M = 1.83, SD = 0.11) categories were used for the negative condition. In the shape condition, participants were engaged in matching the number of vertices of the objects. Dynamic target objects (top) evolved to polygons starting from a circle shape, while probe images (bottom) remained static.

All participants were instructed to use the two-button fibre-optic response pad (Current Design Inc., Philadelphia, PA) with their dominant hand to make a selection (left/right) as soon as they recognized the matching probe. Before the scanning sessions, a short practice run (2 min) was performed to ensure the participants understood the task. In the scanner, we presented the task using video goggles (VisuaStimDigital, Resonance Technology, Northridge, CA) with a resolution of 800 × 600px. After scanning, participants were asked to rate the face triplets for arousal (“How aroused do the faces look?”) and valence (“Do the people feel positive or negative?”) using a continuous slider.

### Computational modeling of the face-matching process

**Fig. 1:**
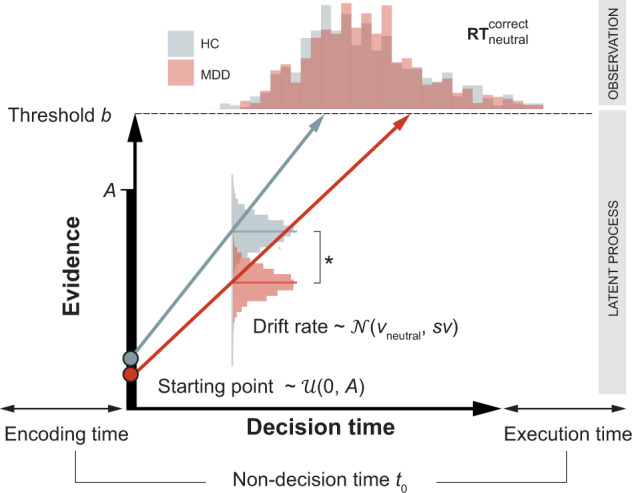
Behavioral parameters. Patients exhibited slower information processing efficiency represented by the lower drift rate during the face matching of neutral faces (Table S4).

The information accumulation process was modeled using the R implementation of a hierarchical linear ballistic accumulator (LBA) model distributed with the Dynamic models of Choice toolbox [45] (DMC, https://osf.io/pbwx8/) by fitting the response data for correct and incorrect trials. Here, the LBA contains two evidence accumulators gathering information for the two possible responses (left or right face). The drift rate parameter *v* quantifies the speed of the evidence accumulation and thus the information processing efficiency. Drift rates for correct and error trials are drawn from a normal distribution with the separately estimated between-trial variability *sv*. To make the model identifiable, typically the *sv* for error responses is fixed at 1. A button press is initiated as soon as one accumulator surpasses the response threshold reflected by the parameter *B*. Lastly, the parameter *t*_0_ captures any effects of non-decision processes (e.g. motor preparation) and the parameter *A* encodes the starting point of the accumulation process (Fig. 1).

We assumed that either the drift rate (i.e. processing efficiency) or the decision threshold (i.e. the evidence required for a response, response caution) are influenced by the different conditions. Thus, we created two models that allowed (a) the drift rate *v* and (b) the threshold parameter *B* to vary as a function of condition. In addition, we created a null model, where both parameters were constant across conditions. In total, three models were fitted to the response data of healthy adolescent controls and participants with MDD separately with Differential Evolution Markov Chain Monte Carlo simulations (DE-MCMC). Thirty-six chains (three times the subject-specific parameters) were used for sampling the posterior distribution of the parameters thinned by keeping only every 10th sample. Initial values for the hierarchical sampling were determined using fixed-effects fits. In the burn-in period, we used a 5% probability of migration for individual and group levels. After burn-in, only the crossover steps of the DE-MCMC algorithm were performed during subsequent sampling. Mixing of chains and stationarity were checked simultaneously by splitting the chains in half and calculating the multivariate potential scale reduction factor [46] $\hat R$ (convergence threshold $\hat R \,<\, 1.1$, Figs. S6, S7).

To determine the best model, we performed model selection using the expected log pointwise predictive density (ELPD). We assessed group differences by comparing the posteriors of the group-level distributions of the best hierarchical model. If the 95% credible interval of the difference distribution of the groups did not include zero, the difference was considered significant.

**Fig. 2:**
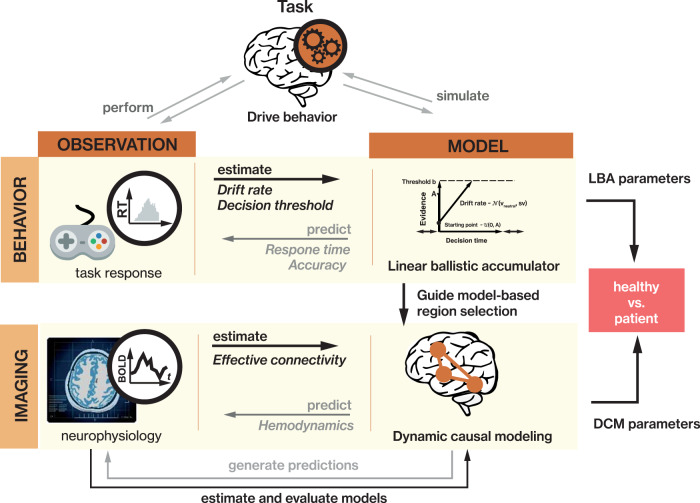
Analysis pipeline. The analysis harnessed generative models of participants’ behavior and neural dynamics. The decision components for the face matching task (e.g. drift rate) were used to identify a network of brain regions from which we were able to derive a mechanistic understanding of behavioral differences. These regions were then used to establish a DCM that describes functional coupling within the network circuitry. Statistical inference was performed separately on LBA and DCM parameters.

We validated the LBA model by simulating data and repeating the raw data analysis on the synthetic data. For this, we simulated data for 2000 subjects (1000 per group) for 20 trials for all conditions and compared the response time distributions with the empricial RTs from our participants (Fig. S5). Then, we randomly selected 33 synthetic controls and 30 patients and mimicked the conventional analysis of log-transformed response times (logRT, see below) by entering them in a linear mixed effects model as in the raw data analysis. The analysis pipeline is presented in Fig. 2.

### Behavioral data analysis

For the behavioral raw data analysis, we performed linear mixed effects model analyses of the logRT, the accuracy and the number of omissions. In addition, we performed a linear mixed effects model analysis on the ratings of valence and arousal for each trial. In all models, condition and group were treated as fixed factors and subject was treated as a random effect. Finally, individual mean ratings were correlated with the subject-wise parameters of the LBA model. The significance level for all statistical tests of the behavioral analyses was *p* < 0.05, two-tailed.

### Neuroimaging data acquisition and preprocessing

MRI recordings were conducted on an Achieva 3T scanner (Philips Medical Systems, Best, the Netherlands) using the manufacturer’s 32-channel head coil array. Functional T2*-weighted image acquisition was performed using a multi-slice echo-planar images (EPI) sequence [335 volumes per session, *TR*=1600ms, *TE* = 35ms, 50 slices, voxel size =2.4 × 2.4 × 2.2 mm^3^, matrix size = 76 × 78*px*, flip angle = 75°, gap = 0.35 mm, SENSE-factor = 2, MB-factor = 2]. Field of view was tilted 15° downwards of AC-PC to improve signal quality in the ventral brain. The first five dummy scans were discarded. A T1-weighted structural scan was acquired for each subject [MP-RAGE, aligned at AC-PC, flip angle = 9°, voxel size = 1.05 × 1.05 × 1.2 mm^3^, field of view = 270 × 253 mm^2^, 170 sagittal slices]. The functional data was first slice-time corrected, then realigned and unwarped using the B0-field map and coregistered to the T1-weighted image. The deformation fields derived from the segmentation of the T1 image were used for normalization to the Montreal Neurological Institute (MNI)-152 template space. The normalized volumes were spatially smoothed using a 6 mm full-width-half-maximum kernel. All steps were conducted in SPM12 (7771). We censored volumes that exceeded a framewise displacement [47] greater than 1 mm using a binary motion scrubbing regressor (% volumes censored per subject *M* = 0.70, *SD* *=* 1.41%).

### Functional MRI data analysis

The task-relevant regions for the dynamic causal modeling (DCM) analysis were identified using a general linear model (GLM) in SPM. We used a combination of the conventional faces vs shapes contrast and a regression of LBA model parameter *v*_*neutral*_ to guide the localization of regions that comprised the task- and disorder-relevant network for the connectivity analysis (see LBA results). The first-level GLM inlcuded an individual regressor for each face condition (negative, positive, neutral) and one for the shapes using the onsets of each trial convolved with the hemodynamic response function. Moreover, six realignment parameters derived from preprocessing and the motion scrubbing regressor were used as nuisance regressors. The fMRI time series data were high-pass filtered with a 128s cut-off and whitened with an AR(1) model. A second-level one-sample *t*-test was used to localize the effects of the task conditions with the scores on the Children’s Depression Inventory as covariate. Linear regression analyses were performed to assess the association between the behavioral model parameters and whole-brain activity. For these analyses we used a cluster-extent threshold to perform family-wise error correction using an uncorrected voxel-wise threshold of *p*_*CDT*_ = 0.001. To derive the cluster size we used Monte Carlo Simulation [48, 49], running 10,000 iterations resulting in a minimum cluster-size of *k* > 440 mm^3^ corresponding to 55 contingent voxels, *p*_*FWE*_ < 0.05. Labels for brain regions are based on the Automated Anatomical Atlas [50].

**Fig. 3:**
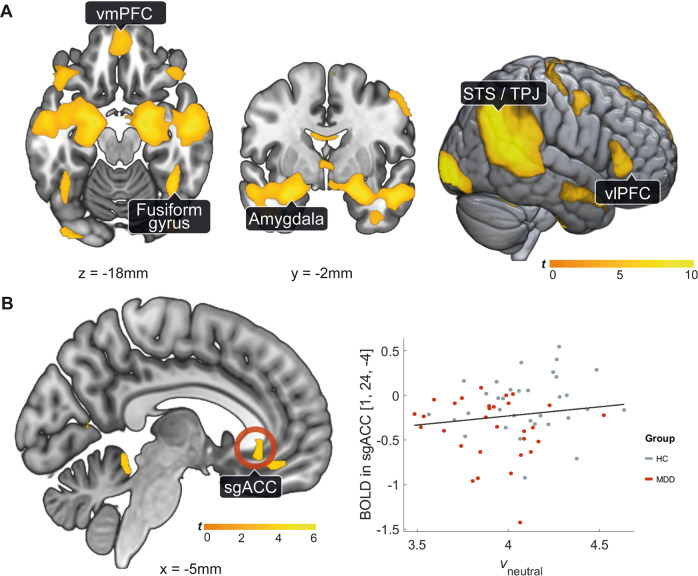
Whole-brain activity analysis. **A** Brain activity for the task (faces-shapes) across both groups. We found activity in the amygdala, the fusiform gyrus, the ventromedial prefrontal cortex (vmPFC), ventrolateral prefrontal cortex (vlPFC), and in a cluster comprising the superior temporal sulcus (STS) and the temporo-parietal junction (TPJ). **B** Brain activity positively associated with the drift rate (i.e. information processing efficiency) during the neutrally/ambiguous valenced dynamic face matching. We found that a slower drift rate is related to a cluster in the subgenual anterior cingulate cortex (sgACC). *p*_FWEc_ < 0.05, *p*_CDT_ < 0.001, *N* = 63.

The selection of the regions for the DCM analsyis was motivated by previous studies of adolescent MDD [25, 33, 35] and our model-based SPM analyses (Fig. 3, Tables S5, S6). We extracted the timeseries from active voxels (*p* < 0.05) within a spherical search volume (*r* = 6 mm) around the group maxima from the faces vs shapes contrast (amygdala [x = 19, y = −8, z = −18 mm]; FFA [x = 41, y = −52, z = −24 mm]; LPFC [x = 53, y = 32, z = 0 mm]) and the linear regression *BOLD*_*neutral*_ ~ *v*_*neutral*_ (sgACC [x = 1; y = 24, z = −4 mm]). Because of the proximity of other active regions, the search volume of the sgACC was additionally constrained with an anatomical mask derived from the meta-analytical coactivation map retrieved from *neurosynth* [51]. We centered the individual spheres around each participant’s maximum, extracted the first eigenvariate of the time course of active voxels (*p* < 0.05), and regressed out motion parameters. The connectivity analysis was constrained to regions in the right hemisphere, since there is evidence that it preferentially processes emotional faces [52, 53] (Figs. S8, S9). For this analysis, we excluded one patient and one control who did not have any significant voxels in the amygdala, and, additionally, one patient who did not disclose their medication status.

As in previous work [36], the mean-centered driving input (all faces) entered the FFA. We set up a fully connected model with modulations of positive and negative valence on all interregional connections. The analysis was conducted within the Parametric Empirical Bayes (PEB) framework where the full DCM model was estimated in an empirical Bayesian inversion scheme for each participant [54]. Group effects on the DCM parameters (i.e. connectivity strengths) were analysed with a PEB model to find group differences between patients and controls within the prefrontal-amygdala network. In brief, the PEB model takes the individuals' connectivity parameters to the second-level using a GLM that partitions the between-subject variability into random effects and explained effects using a design matrix and taking into account the posterior covariance of each parameter. A Bayesian model reduction procedure discarded the model parameters not contributing to the model evidence in a greedy-search by comparing the negative free energies of reduced models [55]. The greedy-search stops, when discarding of any parameter results in a decreased model evidence. Intrinsic connections (*A*-matrix) and contextual modulations (*B*-matrix) were individually analyzed for group differences with selective serotonin reuptake inhibitors (SSRI) medication status, sex, age and handedness as mean-centered covariates. Model parameters were averaged across the best 256 nested models (weighted by their posterior probability) and considered significant when exceeding a 95% posterior probability of being present vs absent based on the model evidence. Finally, we performed a leave-one-out cross-validation (LOOCV, spm_dcm_loo.m) to assess the predictive validity of the dynamic causal model using its accuracy for classification of diagnostic status. This procedure iteratively fits the PEB model to all but the left-out subject and evaluates the posterior belief of the predictor for the unseen participant, i.e. the probability of the diagnostic status. In other words, this procedure swaps the roles of the second-level predictor (diagnosis) and the first-level parameter estimates (connectivity) from the PEB model. When repeating the LOOCV procedure for each subject, a list of probabilities is generated, which can be subsequently used to retrieve the Receiver Operating Characteristic (ROC) curve and the Area Under the Curve (AUC) with 95% confidence bounds across the cross-validation runs (MATLAB perfcurve).

## Results

### Balanced performance measures for both groups

The analysis with a linear mixed-effects model of logRTs showed a significant effect of valence, *F*(3, 4666.9) = 510.07, *p* < 10^−15^. Participants were fastest when presented with positive faces, then negative faces, and slowest in the neutral (i.e. ambiguous emotion) condition. Neither the main effect of group, *F*(1, 61) = 0.26, *p* = 0.615, nor the interaction term, *F*(3, 4666.9) = 0.91, *p* = 0.424, reached significance. The number of incorrect responses showed no main effect of group, *F*(1, 61) = 0.46, *p* = 0.499, and no significant group-by-condition interaction, *F*(3, 183) = 0.04, *p* = 0.989. Across conditions, however, there was a significant main effect, *F*(3, 183) = 129.94, *p* < 10^−15^. Participants were more accurate when presented with negative or positive faces, or shapes, compared to neutral faces. The number of response omissions did not show a significant effect of group, *F*(1,61) = 1.23, *p* = 0.272, nor a significant group-by-condition interaction, *F*(3,183) = 0.38, *p* = 0.766. There was a significant main effect of condition, *F*(3,183) = 38.26, *p* < 10^−15^, with neutral faces and shapes having more response omissions than positive and negative faces. These results suggest that the face-matching task was equally difficult for both groups. Results are summarized in Table S1 and Fig. S2.

### Biased arousal and valence ratings in MDD

Arousal ratings showed a significant condition-by-group interaction, *F*(2,3713) = 22.70, *p* < 10^−9^, with patients’ arousal rating for negative faces being higher than controls’. We also found a significant effect of condition, *F*(2,3713) = 565.60, *p* < 10^−15^, with arousal ratings increasing from neutral, positive and negative faces, but no main effect of group, *F*(1,61) = 0.002, *p* = 0.97. Analysis of valence ratings revealed a condition-by-group interaction, *F*(2,3713) = 4.34, *p* = 0.013, with patients having lower valence ratings for positive faces. The significant main effect of condition, *F*(2,3713) = 2491.02, *p* < 10^−16^, showed that ratings increasing from negative to neutral, and from neutral to positive (Fig. S2). The main effect of group was not significant, *F*(1,61) = 1.209, *p* = 0.27. Lastly, the valence rating of neutral faces was negatively associated with the drift rate parameter *v*_*neutral*_, *r*(59) = −0.395, *p* = 0.002 (Fig. S3).

### Decreased face processing efficiency in MDD

We estimated the parameters of the LBA model using response time and accuracy data. First, we were interested in how the parameters were modulated by our different conditions (positive, negative, neutral faces, and shapes). Model *M*_*v*_, where the drift rate was allowed to vary by condition, accounted for the data best in both groups (Table S2). Subsequently, we tested whether the parameters in the winning model *M*_*v*_ revealed any group differences. We found evidence that during matching of neutral faces, participants with MDD exhibited slower information accumulation compared to healthy controls, ∆*v*_*neutral*_ = 0.14, 95% *HPDI* (Highest Posterior Density Interval) [0.03, 0.24] (Table S4, Fig. 1). Furthermore, information accumulation also tended to be slower for positive, ∆*v*_*positive*_ = 0.22, [−0.03, 0.47], and negative faces, ∆*v*_*negative*_ = 0.19, [−0.01, 0.40], however these effects missed the significance level marginally (Fig. S4). In contrast, we found only little evidence that drift rate for shapes, ∆*v*_*shapes*_ = 0.05, [−0.13, 0.21], overall decision threshold, ∆*B* = −0.20, [−0.67, 0.24], bias ∆*A* = 0.26, [−0.30, 0.88], or non-decision time, ∆*t*_0_ = 0.03, [−0.06, 0.13], differed between patients and controls.

### Posterior predictive checks of the behavioral model

The analysis of the synthetic data using a linear mixed-effects model of logRTs showed great agreement with the results from the empirical data. The synthetic data reproduced the significant effect of valence, *F*(3, 4971) = 479.23, *p* < 10^−16^. Posthoc comparisons corroborated that the model captured the fastest responses for positive faces and the slowest responses for neutral faces. As in the main analysis, the effect of group, *F*(1, 61) = 1.42, *p* = 0.238, and the group-by-condition interaction, *F*(3, 4971) = 0.77, *p* = 0.514, were not significant. Therefore, we can conclude that the LBA model was able to capture the effects in our data (Figs. S5–S7).

### DCM model structure for emotional face processing

The goal of the DCM analysis was to identify the brain network dynamics related to the aberrant information accumulation in MDD patients. The task contrast (faces vs shapes) revealed expected activation differences in brain areas commonly reported in face-matching and emotion processing paradigms (Table S5) [36, 56]. The group comparison for the task contrast revealed no significant clusters. The rate of evidence accumulation encoded by the drift rate *v*_*neutral*_ (Fig. 3B, Table S6) varied as a function of the sgACC activity during neutrally (i.e. ambiguously) valenced face matching.

### FFA – LPFC – sgACC pathway associated with depressive status

**Fig. 4:**
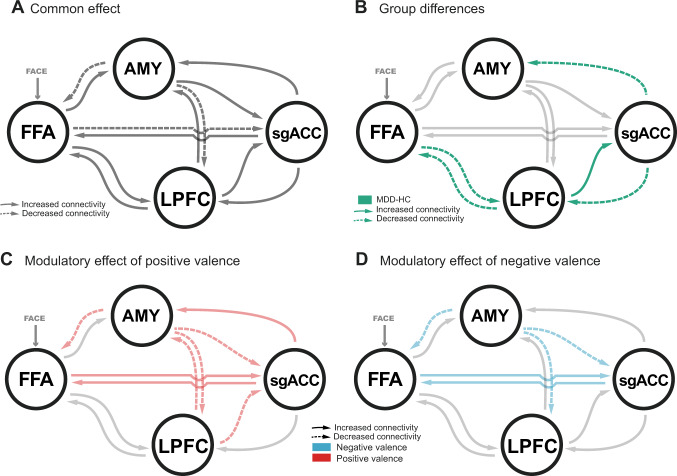
DCM analysis of the prefrontal-amygdala network. **A** The common effect represents the overall model structure for the baseline (neutral faces) across all participants. **B** Group differences were primarily found in the bidirectional cortical pathway FFA-LPFC-sgACC for the average connectivity across conditions. In addition, connectivity between sgACC and the amygdala was decreased in patients. **C**, **D** Efferent connectivity from the amygdala was modulated by positive (**C**) and negative (**D**) valence. In addition, processing of positive valence was associated with altered LPFC—amygdala connectivity and the LPFC—sgACC—amygdala pathway. Detailed results are reported in Table S7. AMY amygdala, FFA fusiform face area, HC healthy controls, LPFC lateral prefrontal cortex, MDD major depressive disorder, sgACC subgenual anterior cingulate cortex.

The overall model structure of the DCM included all connections within the network, i.e. each connection within the network contributed significantly to the model evidence. Diagnostic status had a significant effect on the coupling strength along the cortical FFA – LPFC – sgACC pathway (Fig. 4, Table S7). Participants with a clinical diagnosis of MDD showed decreased bi-directional connectivity between the FFA and the LPFC (expected values = 0.033 Hz and −0.085 Hz, PP = 1.00). Furthermore, the efferent connectivity from the sgACC to the LPFC was decreased (expected value = −0.036 Hz, PP = 1.00), whereas the coupling from the LPFC to the sgACC was heightend (expected value = 0.054 Hz, PP = 1.00). In addition, we found decreased connectivity between sgACC and amygdala in patients during face processing (expected value = −0.035 Hz, PP = 1.00). LOOCV demonstrated that connectivity within the pathway derived form the group comparison was able to predict individual group labels, while controlling for all covariates (AUC = 0.71, 95% CI [0.56 0.84], Fig. S10). Performing LOOCV on individual connections showed that the connections from LPFC to sgACC (AUC = 0.67, 95% CI [0.51 0.78]) and FFA to LPFC (AUC = 0.65, 95% CI [0.51 0.79]) were the most predictive.

### Selective serotonin reuptake inhibitor use affects sgACC function

As the majority of our patients (*n* = 18) received SSRIs as treatment during the participation in our study, we included it as covariate in our model and assessed its effect on connectivity. We found that patients with SSRI intake had a decreased self-connection in the sgACC (expected value = −0.145 Hz, PP = 1.00, Table S7). The lower this parameter, the more readily the region is excited by the network inputs, i.e. SSRI intake was related to increased sgACC input sensitivity.

### Valence-dependent prefrontal-amygdala coupling

The efferent amygdalar connectivity pattern changed specifically for emotional context (B-matrix) compared to the average connectivity across conditions (A-matrix). In particular, we found that during processing of positive and negative faces, amygdala-FFA (pos: expected value = −0.434 Hz, PP = 1.00; neg: expected value = −0.26 Hz, PP = 1.00;) and amygdala-LPFC (pos: expected value = −0.121 Hz, PP = 0.97; neg: expected value = −0.174 Hz, PP = 1.00) connectivity showed a stronger negative coupling (Table S7, Fig. 4). Functional coupling from amygdala to sgACC decreased during emotional contexts (pos: expected value = −0.308 Hz, PP = 1.00; neg: expected value = −0.127 Hz, PP = 0.97), whereas the connectivity from FFA to sgACC increased (pos: expected value = 0.228 Hz, PP = 1.00; neg: expected value = 0.095 Hz, PP = 1.00). Only for positive faces, we found a decrease in connectivity between LPFC and sgACC (expected value = −0.186 Hz, PP = 1.00) and strengthened coupling from sgACC to amygdala (expected value = 0.291 Hz, PP = 1.00). However, none of the contextual modulations was different between patients and controls.

## Discussion

The assessment of emotional face processing using a model-based neuroimaging approach was able to identify cognitive and neural mechanisms that characterize adolescent MDD. Our findings show that the distributed alterations in brain connectivity within the prefrontal-amygdala network of adolescents with MDD are strongly related to decreased processing efficiency of facial affect.

On the behavioral level, our data show that facial affect was associated primarily with information processing efficiency (i.e. task difficulty), and not the response threshold (i.e. quantified caution) of participants. Moreover, decreased face processing efficiency in MDD was specifically evident in the most ambiguous valence condition, i.e. neutral faces. Thus, we showed that evidence accumulation does not only depend on the contextual factor of facial affect (i.e. valence), but is also highly sensitive for group differences of more subtle, ambiguous facial expressions. Perceptual differences were also reflected in participants’ ratings. Patients showed biases for negative faces in the form of increased arousal and positive faces in the form of decreased valence. This suggests that participants with MDD might struggle with decoding facial emotions, because they are slower and less accurate and their perception is biased. Our results provide a novel mechanistic explanation for the negatively biased evaluation process of emotion in adolescent [57] and adult MDD [11, 58].

Based on the proposition that the ventral-affective brain network is vulnerable to such negatively biased processing in adolescence [13], we sought to identify aspects of the functional architecture of the prefrontal-amygdala circuitry that could explain face processing difficulties in MDD. Combining the behavioral model with fMRI data revealed that a slower evidence accumulation process was associated with stronger deactivation in the sgACC. This effect was explained by altered coupling along the FFA-LPFC-sgACC pathway in patients with MDD. This finding is in line with earlier studies in adolescents with and at risk of MDD [25, 26, 33] and adult MDD [24, 59, 60] that implicated functional abnormalities of the sgACC and the FFA in the etiology of depression. Specifically, dysfunction of the FFA [38] and increased functional connectivity between FFA and sgACC [25] have been reported in adolescent MDD during emotion processing. However, beyond that, our findings suggest a disrupted top-down connectivity between sgACC and amygdala for face stimuli. Altered coupling between perceptual, cognitive and affective regions is likely to contribute to the decreased processing efficiency and thus to poor evidence sampling from facial expressions during the task. Within the prefrontal-amygdala circuitry, the sgACC acts as gatekeeper between the cognitive prefrontal and limbic systems [61] and is in a position to adjust sampling of the sensory evidence [62]. It encodes precise predictions about changes in the environment and anticipating emotional situations that eventually generate a bodily response [62, 63]. In a natural environment, facial expressions represent noisy sensory information that can be used to deduce a person’s intention [64]. Therefore, any disruption of sgACC engagement during ambiguous interpersonal situations might yield maladaptive autonomous regulation resulting in inappropriate emotional responses [65]. An important next step will be to clarify how differences in top-down vs bottom-up connectivity between the amygdala and sgACC in our study compared to previous work [28, 33] are related to specific aspects of the (a) employed stimuli (e.g. fearful vs. sad faces) or (b) if they represent distinct subtypes of depression. Importantly, the MDD-specific coupling was consistent across all facial emotions, that is there was no evidence for group differences in valence-dependent modulations, suggesting general rather than valence-specific emotion processing aberrations in this network.

Across healthy controls and patients, we observed an emotion-dependent change of prefrontal-amygdala connectivity for negative and positive faces. Specifically, the excitatory coupling from amygdala to sgACC became inhibited. This is consistent with previous studies in adults [66, 67], and underscores the importance of coupling between these regions to process emotional stimuli in concert. Coupling from the amygdala to the visual FFA [68] and the evaluative LPFC [69] became even more inhibitory for positive and negative faces. Given that these connections have been previously described as excitatory in healthy adults [36, 70, 71], we hypothesize that these functional connections are subject to a developmental change, consistent with maturational fine-tuning of brain connectivity across adolescence [72, 73]. Our results support this notion by showing that connectivity between these regions increased with age, and thus, the affective component might be dampened in favor of increased cognitive control in a more mature state towards adulthood. Across all participants, LPFC-sgACC-amygdala coupling was modulated stronger by positive faces compared to neutral and negative faces. The role of prefrontal-amygdala interactions during emotion processing, significance detection and the resolution of uncertainty is well established [74]. In contrast, during the negative faces condition there were no deviations from the average connectivity along this pathway. This might be related to the fact that in our dynamic face and shape-matching task, the conditions other than positive valence represent an immanent situational uncertainty or ambiguity with potential demand for action. Thus, the LPFC-sgACC-amygdala pathway might be engaged for an orientation response to resolve this uncertainty [75].

Lastly, more than half of the patients included in this study were treated with SSRIs. We found that patients receiving SSRIs had increased sgACC input sensitivity. Functional changes of sgACC in response to SSRI treatment have been reported in another study of adolescent MDD [22] and could reflect a potential neural mechanism on how SSRIs alter the functional brain circuit to help ameliorate depressive symptoms in the long term. Previous studies using emotion processing paradigms have shown that prefrontal-amygdala connectivity might allow treatment prediction in MDD. Jamieson et al. [34] investigated interactions between amygdala and ventromedial and dorsolateral PFC, respectively, and showed that connectivity between amygdala and PFC during implicit emotion processing have been predictive for subsequent treatment response to combined cognitive-behavioral therapy and SSRI in youth. Also in adult MDD, pre-treatment brain connectivity between amygdala and dorsal ACC has been implicated in predicting treatment response [76]. Although our results are exploratory, we believe that this finding blends in with previous work and may guide future research of suitable biomarkers for the prediction of SSRI treatment response.

Using behavioral models allows to disentangle distinct components of cognitive processing and to identify their neural correlates. The combination of cognitive modeling and neuroimaging provides a refined understanding of disease mechanisms in depression and is able to advance our knowledge of *how* information processing goes awry [77]. For instance, rather than a dysfunction of one individual brain region, our results suggest a dysbalance of a functional brain network. This underscores the utility of new methodological developments in the field of computational psychiatry that allow for a comprehensive characterization of cognitive and neural mechanism which can facilitate the translation of research into novel clinical tools [77].

Although our study offers compelling mechanistic insights into altered emotion processing in adolescents with MDD, our data should be interpreted within the given limitations. Longitudinal designs will be crucial to gain causal insights of clinical trajectories in the future. Furthermore, the modest sample size reflects the recruitment challenges for the study population. Third, in the current task, our participants achieved a high ratio of correct/error trials for positive and negative faces. Altough in the Dynamic Models of Choice framework this can be at least partially accounted for by including conditions with a sufficient error rate [45], this might have led to poorer sensitivity to reveal group differences in the positive and negative face conditions than in the neutral condition. Finally, due to our study design the origin of the SSRI treatment effect remains unclear since our evidence is only correlative. It will be important for future research to investigate whether this effect is related to a normalization or compensation in patients.

To summarize, this work presented an analytic approach to study the cognitive and neural mechanisms of emotion processing in adolescent MDD that revealed diminished cognitive efficiency and altered function of brain circuits supporting emotion processing. Thus, the current work provides novel insights into impairments of emotion processing and significantly advances our understanding how altered emotion processing is affected in adolescent MDD. Ultimately, this holds promise to improve the development of targeted interventions with psychotherapy and pharmacotherapy, and potentially also novel neurofeedback approaches [78] aiming at cognitive bias modification in patients or those at-risk [79].

## Supplementary information

Supplemental material

## Data Availability

The relevant codes used to generate results are available from the authors on request, subject to compliance with the requirements of the ethics committee of the canton of Zurich.
