# Supplementary material for: Deficient prefrontal-amygdalar connectivity underlies inefficient face processing in adolescent major depressive disorder
Source: Transl Psychiatry. 2022 May 10;12:195. doi: 10.1038/s41398-022-01955-5 (PMC9090758; doi:10.1038/s41398-022-01955-5)
Supplement: Supplementary file 1 — Supplemental material [file 41398_2022_1955_MOESM1_ESM.docx]

# Supplementary Tables

| **Table S1**. Descriptive summary statistics of response data for all conditions in both groups. | | | | | |
| --- | --- | --- | --- | --- | --- |
|  | | HC | MDD | Test statistic | *p*-value |
| Response time (s) | Negative | 2.42 (0.33) | 2.45 (0.32) | *t*(61) = -0.403 | .688 |
|  | Positive | 2.21 (0.36) | 2.24 (0.30) | *t*(61) = -0.307 | .760 |
|  | Neutral | 2.93 (0.22) | 3.01 (0.22) | *t*(61) = -1.387 | .171 |
|  | Shapes | 2.90 (0.29) | 2.89 (0.31) | *t*(61) = 0.111 | .912 |
| Accuracy (%) | Negative | 97.6 (3.8) | 96.7 (4.0) | *t*(61) = 0.927 | .358 |
|  | Positive | 98.8 (2.8) | 98.2 (4.3) | *t*(61) = 0.691 | .493 |
|  | Neutral | 72.6 (18.0) | 71.5 (14.4) | *t*(61) = 0.260 | .796 |
|  | Shapes | 77.7 (10.2) | 76.0 (12.8) | *t*(61) = 0.595 | .554 |
| Data is shown in mean (SD). Tests are performed on the means of each subject.  Abbreviations: HC, healthy controls; MDD, major depressive disorder. | | | | | |

| **Table S2.** Behavioral model comparison. | | | | |
| --- | --- | --- | --- | --- |
|  |  | $\mathcal{M}_{\boldsymbol{0}}$ | $\mathcal{M}_{\boldsymbol{v}}$ | $\mathcal{M}_{\boldsymbol{B}}$ |
| ELPD | HC | 718 | 1154* | 1133 |
|  | MDD | 633 | 1019* | 1006 |
| Abbreviations: ELPD, expected log predictive density; HC, healthy controls; MDD, major depressive disorder. $\mathcal{M}_{\boldsymbol{0}}$=null model, $\mathcal{M}_{\boldsymbol{v}}$=model with varying drift rate, $\mathcal{M}_{\boldsymbol{B}}$=model with varying decision threshold. Asterisk indicates the best model. | | | | |

| Table S3. Priors of the group-level parameters of the Linear Ballistic Accumulator model | |
| --- | --- |
| $\boldsymbol{A}$ | $\mathcal{N}\left( \mu=1,\sigma^{2}=1 \right) \vert\mu>0$ |
| $\boldsymbol{B}$ | $\mathcal{N}\left( \mu=1,\sigma^{2}=1 \right) \vert\mu>0$ |
| $\boldsymbol{v}$ | $\mathcal{N}\left( \mu=2,\sigma^{2}=3^{2} \right)$ |
| $\boldsymbol{v}^{\boldsymbol{error}}$ | $\mathcal{N}\left( \mu=1,\sigma^{2}=3^{2} \right)$ |
| $\boldsymbol{sv}$ | $\mathcal{N}\left( \mu=1,\sigma^{2}=1 \right) \vert\mu>0$ |
| $\boldsymbol{t}_{\boldsymbol{0}}$ | $\mathcal{N}\left( \mu=1,\sigma^{2}={.25}^{2} \right) \vert\mu>.1$ |
| Abbreviations: *A* = Starting point, *B* = Decision threshold-*A*, *v* = drift rate, *sv* = standard deviation of drift rate, *t*_0_ = non-decision time | |

| Table S4. Parameter estimates of the LBA model and comparison between groups | | | |
| --- | --- | --- | --- |
|  | HC | MDD | HC-MDD |
| $\boldsymbol{A}$ | 5.72 [5.31, 6.09] | 5.45 [4.98, 5.86] | 0.26 [-0.30, 0.88] |
| $\boldsymbol{B}$ | 5.55 [5.27, 5.83] | 5.75 [5.40, 6.10] | -0.20 [-0.67, 0.24] |
| $\boldsymbol{v}_{\boldsymbol{negative}}$ | 4.10 [3.96, 4.24] | 3.91 [3.77, 4.06] | 0.19 [-0.01, 0.40] |
| $\boldsymbol{v}_{\boldsymbol{neutral}}$ | 3.15 [3.08, 3.23] | 3.01 [2.94, 3.09] | 0.14 [0.03, 0.24] * |
| $\boldsymbol{v}_{\boldsymbol{positive}}$ | 4.50 [4.31, 4.70] | 4.29 [4.12, 4.44] | 0.22 [-0.03, 0.47] |
| $\boldsymbol{v}_{\boldsymbol{shapes}}$ | 3.28 [3.17, 3.39] | 3.23 [3.10, 3.36] | 0.05 [-0.13, 0.21] |
| $\boldsymbol{v}_{\boldsymbol{negative}}^{\boldsymbol{error}}$ | -0.53 [-1.22, 0.07] | -0.34 [-0.99, 0.31] | -0.19 [-1.07, 0.75] |
| $\boldsymbol{v}_{\boldsymbol{neutral}}^{\boldsymbol{error}}$ | 1.87 [1.59, 2.16] | 1.76 [1.56, 1.95] | 0.11 [-0.25, 0.45] |
| $\boldsymbol{v}_{\boldsymbol{positive}}^{\boldsymbol{error}}$ | -0.53 [-1.25, 0.14] | -1.16 [-1.91, -0.43] | 0.63 [-0.35, 1.67] |
| $\boldsymbol{v}_{\boldsymbol{shapes}}^{\boldsymbol{error}}$ | 1.59 [1.41, 1.76] | 1.61 [1.44, 1.79] | -0.02 [-0.27, 0.23] |
| $\boldsymbol{sv}$ | 0.54 [0.50, 0.58] | 0.52 [0.45, 0.57] | 0.03 [-0.05, 0.10] |
| $\boldsymbol{t}_{\boldsymbol{0}}$ | 0.17 [0.10, 0.25] | 0.14 [0.10, 0.18] | 0.03 [-0.06, 0.13] |
| Means of hyperparameters (95% credible interval) within each group and their difference.  Asterisk indicates a significant difference. | | | |

| **Table S5. Task effect (faces > shapes) across both groups** | | | | | | |
| --- | --- | --- | --- | --- | --- | --- |
|  | MNI coordinates [mm] | | |  |  | |
| Brain region | x | y | z | k | | Peak Z |
| Occipital_Inf_R | 27 | -96 | -4 | 866 | | > 8 |
| Occipital_Inf_L | -23 | -98 | -6 | 637 | | > 8 |
| Cingulate_Mid_R | 5 | -52 | 34 | 2222 | | > 8 |
| Angular_R | 59 | -66 | 26 | 4774 | | > 8 |
| Angular_L | -51 | -64 | 50 | 4447 | | > 8 |
| Amygdala_R | 19 | -8 | -18 | 526 | | > 8 |
| Amygdala_L | -19 | -8 | -16 | 535 | | > 8 |
| Fusiform_R | 41 | -52 | 22 | 139 | | > 8 |
| Fusiform_L | -41 | -52 | -24 | 84 | | 7.30 |
| Frontal_Inf_Tri_L | -53 | 26 | 0 | 424 | | 7.27 |
| Frontal_Sup_2_L | -15 | 44 | 44 | 145 | | 6.97 |
| NA | -11 | -26 | 24 | 175 | | 6.84 |
| Frontal_Med_Orb_L | 1 | 52 | 14 | 125 | | 6.44 |
| Frontal_Inf_Tri_R | 53 | 32 | 0 | 89 | | 6.22 |
| Rolandic_Oper_L | -39 | -18 | 20 | 96 | | 6.06 |
| Frontal_Sup_2_L | -13 | 58 | 18 | 362 | | 6.04 |
| NA | 13 | -32 | 22 | 56 | | 6.03 |
| Frontal_Sup_2_R | 15 | 36 | 50 | 69 | | 5.93 |
| Paracentral_Lobule_R | 3 | -30 | 60 | 63 | | 5.76 |
| Significance level at whole-brain correction pFWE < 0.05, minimum cluster size k > 55.  Abbreviations: k, cluster size; R, right; L, left; NA, not available; MNI, Montreal Neurological Institute. | | | | | | |

| **Table S6. Positive associations between the drift rates and brain activity** | | | | | | |
| --- | --- | --- | --- | --- | --- | --- |
|  | MNI coordinates [mm] | | |  |  | |
| Brain region | x | y | z | k | | Peak Z |
| *v*_neutral_ | | | | | | |
| Precentral_R | 53 | -2 | 22 | 305 | | 4.54 |
| Lingual_L | -11 | -42 | -10 | 136 | | 4.43 |
| ACC_Sub_L | 1 | 24 | -4 | 81 | | 4.38 |
| Frontal_Med_Orb_L | -9 | 38 | -14 | 103 | | 4.33 |
| Cerebellum_6_L | -19 | -60 | -20 | 70 | | 4.00 |
| Cuneus_R | 19 | -72 | 26 | 89 | | 4.00 |
| Calcarine_L | -11 | -68 | 6 | 96 | | 3.94 |
| Calcarine_R | 19 | -62 | 6 | 95 | | 3.78 |
| *v*_positive_ | | | | | | |
| Orbital_Inf_L | -53 | 80 | -6 | 85 | | 5.26 |
| Postcentral_L | -51 | -20 | 42 | 218 | | 4.29 |
| Temporal_Sup_R | 67 | -36 | 20 | 148 | | 4.23 |
| Temporal_Inf_R | 55 | -72 | -8 | 85 | | 4.21 |
| Temporal_Mid_R | 55 | -32 | -4 | 130 | | 4.16 |
| Precentral_R | 63 | 10 | 28 | 230 | | 4.12 |
| Significance level at whole-brain cluster-level pFWEc < 0.05.  Abbreviations: k, cluster size; R, right; L, left; MNI, Montreal Neurological Institute. | | | | | | |

| **Table S7. Connectivity strength (posterior probability) during face-matching obtained by Bayesian model averaging of PEB model parameters.** | | | | | | |
| --- | --- | --- | --- | --- | --- | --- |
| **Connection type** | **Common** | **MDD** | **SSRI** | **Age** | **Sex** | **Handed.** |
| **Endogenous parameters** | | | |  |  |  |
| FFA→AMY | 0.118 (1) | - | - | - | - | - |
| FFA→LPFC | 0.226 (1) | -0.033 (1) | - | - | - | - |
| FFA→sgACC | -0.202 (1) | - | - | - | - | 0.048 (1) |
| AMY→FFA | -0.687 (1) | - | - | 0.045 (1) | - | - |
| AMY→LPFC | -0.345 (1) | - | - | 0.026 (1) | 0.055 (1) | - |
| AMY→sgACC | 0.086 (1) | - | - | - | - | - |
| LPFC→AMY | 0.084 (1) | - | - | - | - | 0.064 (1) |
| LPFC→FFA | 0.270 (1) | -0.085 (1) | - | - | -0.065 (1) | 0.140 (1) |
| LPFC→sgACC | 0.339 (1) | 0.054 (1) | - | - | - | - |
| sgACC→AMY | 0.325 (1) | -0.035 (1) | - | - | - | - |
| sgACC→FFA | 1.251 (1) | - | - | - | - | - |
| sgACC→LPFC | 0.260 (1) | -0.036 (1) | - | - | - | - |
| **Self-inhibition parameters** | | | | | | |
| AMY→AMY | -0.653 (1) | - | - | -0.059 (1) | -0.146 (1) | - |
| FFA→FFA | 0.148 (1) | **-** | - | - | - | 0.203 (1) |
| LPFC→LPFC | -0.799 (1) | -0.139 (1) | - | - | - | - |
| sgACC→sgACC | -0.651 (1) | - | -0.145 (1) | 0.063 (1) | - | - |
| **Modulatory parameters** | | | | | | |
| FFA→AMY, pos. | - | - | - | - | - | - |
| FFA→LPFC, pos. | - | - | - | - | - | - |
| FFA→sgACC, pos. | 0.228 (1) | - | - | - | - | - |
| AMY→FFA, pos. | -0.434 (1) | - | - | - | - | - |
| AMY→LPFC, pos. | -0.121 (0.97) | - | - | - | - | - |
| AMY→sgACC, pos. | -0.308 (1) | - | - | - | - | - |
| LPFC→AMY, pos. | -0.233 (1) | - | - | - | - | - |
| LPFC→FFA, pos. | 0.146 (0.90) | - | - | - | - | - |
| LPFC→sgACC, pos. | -0.186 (1) | - | - | - | - | - |
| sgACC→AMY, pos. | 0.291 (1) | - | - | - | - | - |
| sgACC→FFA, pos. | 0.636 (1) | - | - | - | - | - |
| sgACC→LPFC, pos. | - | - | - | - | - | - |
| FFA→AMY, neg. | - | - | - | - | - | - |
| FFA→LPFC, neg. | 0.051 (0.72) | - | - | - | - | - |
| FFA→sgACC, neg. | 0.095 (0.98) | - | - | - | - | - |
| AMY→FFA, neg. | -0.260 (1) | - | - | - | - | - |
| AMY→LPFC, neg. | -0.174 (1) | - | - | - | - | - |
| AMY→sgACC, neg. | -0.127 (0.97) | - | - | - | - | - |
| LPFC→AMY, neg. | -0.085 (0.89) | - | - | - | - | - |
| LPFC→FFA, neg. | - | - | - | - | - | - |
| LPFC→sgACC, neg. | - | - | - | - | - | - |
| sgACC→AMY, neg. | - | - | - | - | - | - |
| sgACC→FFA, neg. | 0.291 (1) | - | - | - | - | - |
| sgACC→LPFC, neg. | - | - | - | - | - | - |
| **Input parameter** | | | |  |  |  |
| All faces→FFA | 0.313 (1) | - | - | - | - |  |
| Between-region connections are in units of Hz. Self-inhibition parameters, where the source and target are the same, are the log of scaling parameters that multiply up or down the default value −0.5Hz. Posterior probabilities are given in the brackets. This table includes all parameters of the 256 best models during Bayesian model averaging. *n* = 60. AMY, amygdala; FFA, fusiform face area; LPFC, lateral prefrontal cortex; sgACC, subgenual anterior cingulate cortex; pos., positive faces; neg., negative faces. | | | | | | |

# Supplementary Figures


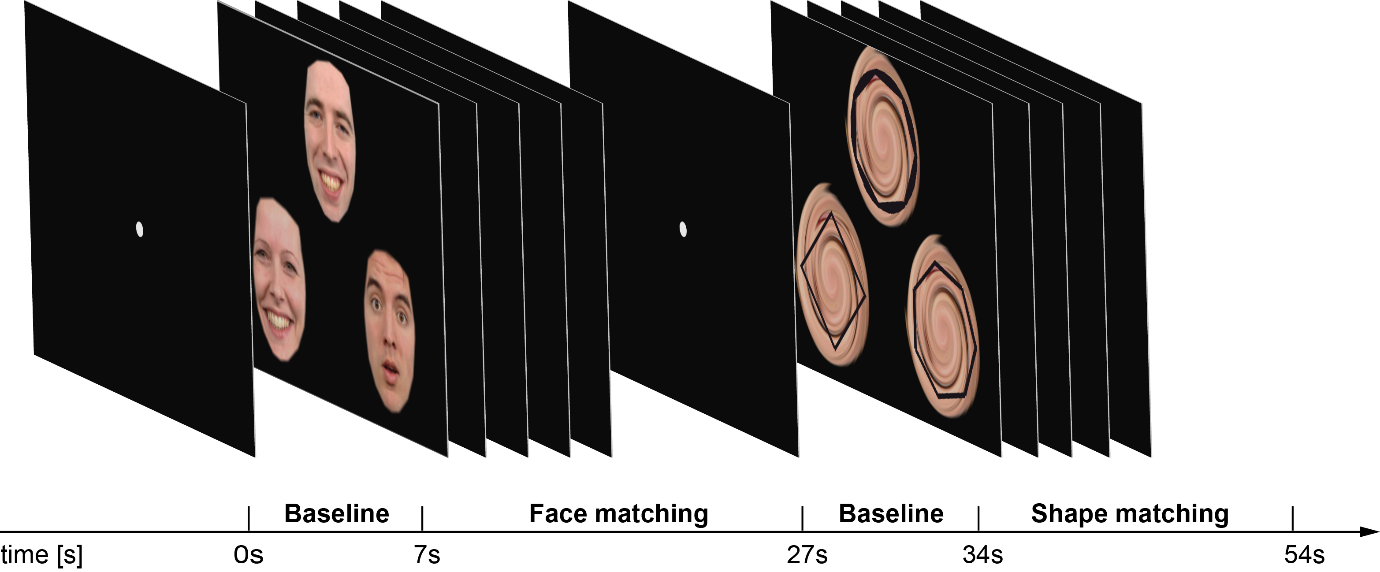


**Figure S1**. The dynamic face and shape-matching task examines the emotion processing circuitry [^1^](#_ENREF_1). Here, the task comprised different conditions with face stimuli varying in valence (positive, negative, neutral) and shapes. Participants were instructed to respond via button press as soon as they identified the matching emotion or shape. Per condition, we presented 5 trials per block and 4 blocks in total, i.e. 20 trials for each of the four conditions.


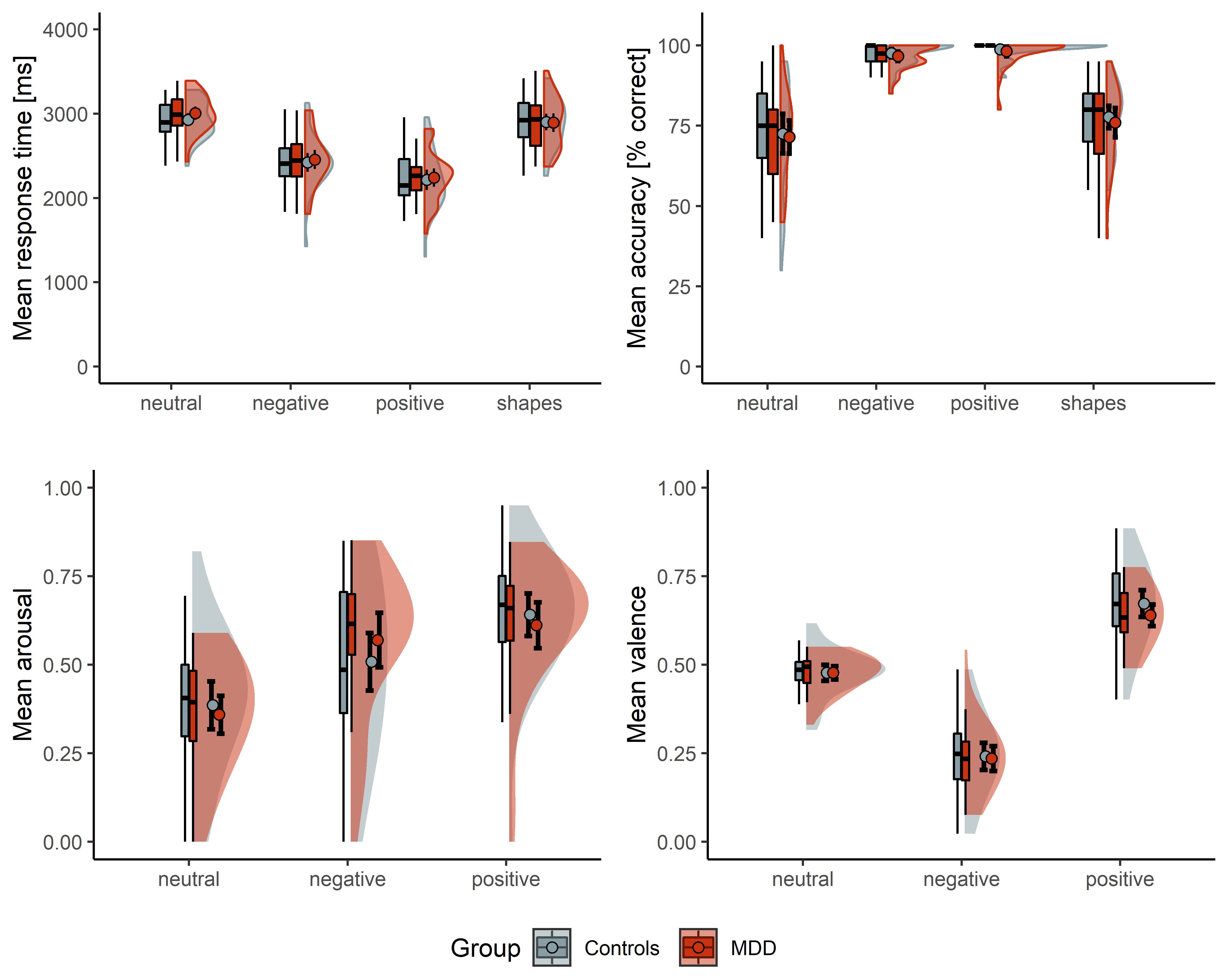


**Figure S2**. Summary of behavioral data. Colored dots represent the group means, black bars the 95% confidence intervals.


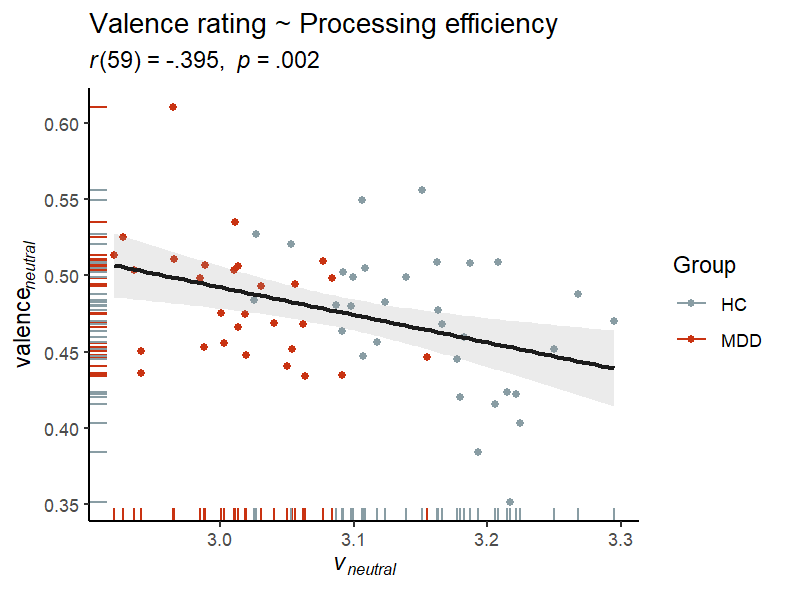


**Figure S3**. Processing efficiency of neutral faces was negatively correlated with valence ratings.


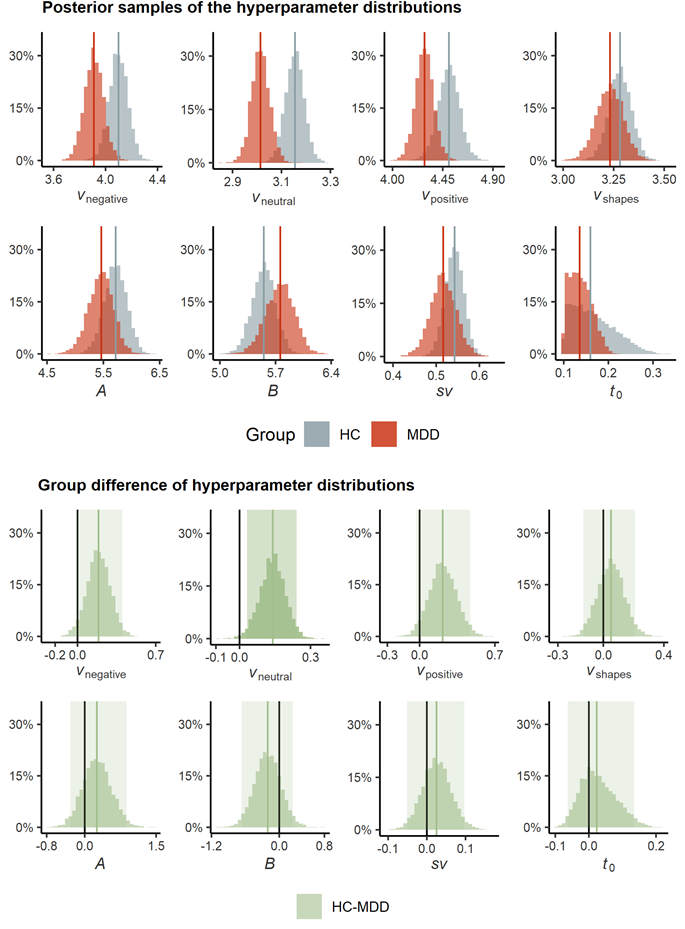


**Figure S4**. Summary of the hyperparameters of the linear ballistic accumulator model.


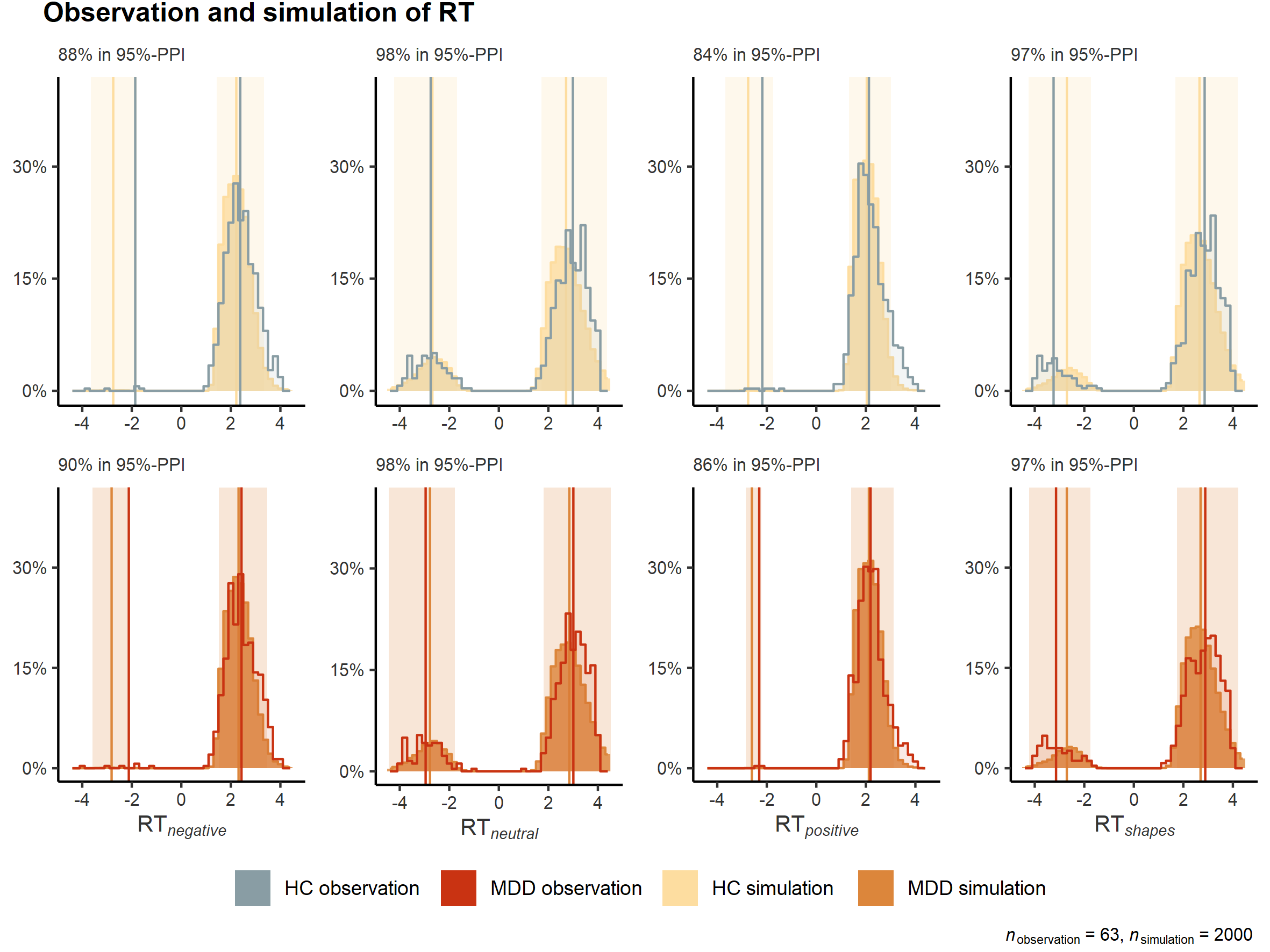


**Figure S5**. Posterior predictive checks. RT distributions for observed and simulated responses: positive values represent correct trials, error trials are negative. Simulation of 1000 participants per group performing our task showed, that the results could be replicated well. As expected, model parameters for task conditions with a low error rate were more difficult to estimate and simulated data was scarce. However, all observed medians were within the 95% posterior predictive interval (shaded) indicating a good concordance.


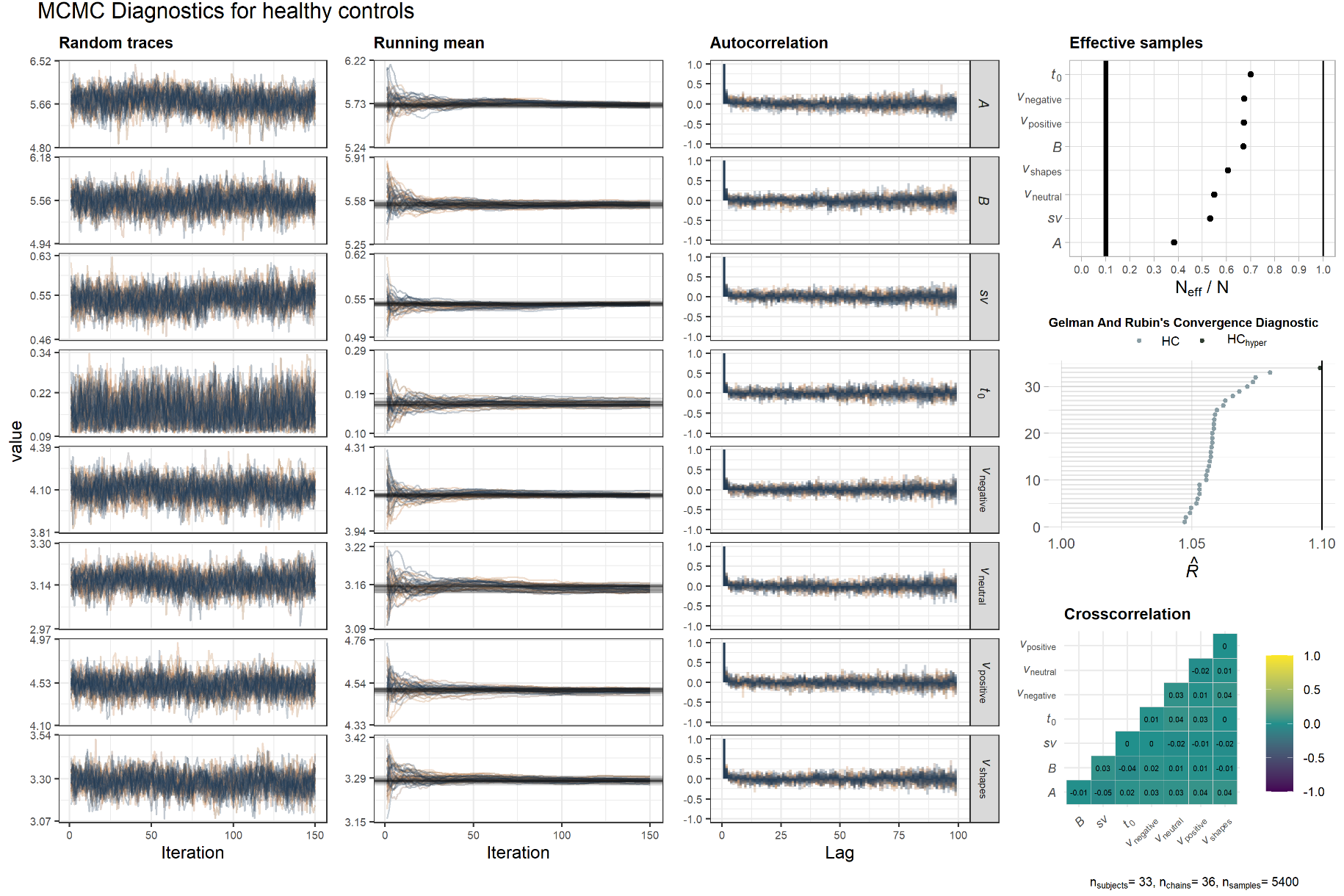


**Figure S6**. Diagnostics for MCMC in healthy controls. After thinning, 5400 samples retained per parameter. Here, we show the traces of the retained samples, the running mean and the autocorrelation.


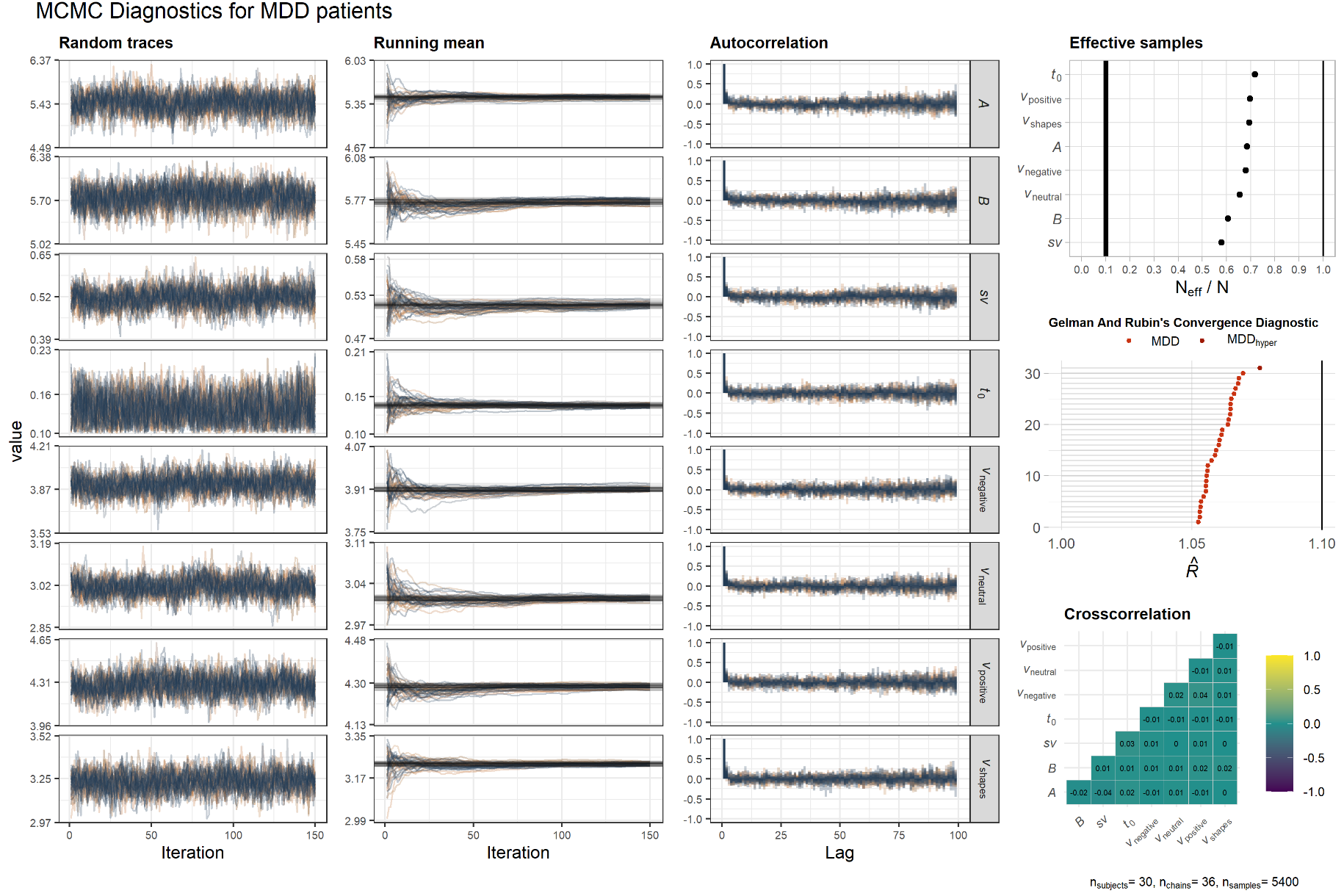


**Figure S7**. Diagnostics for MCMC in MDD patients. As in healthy controls, MCMC diagnostic showed good convergence of the model parameters.


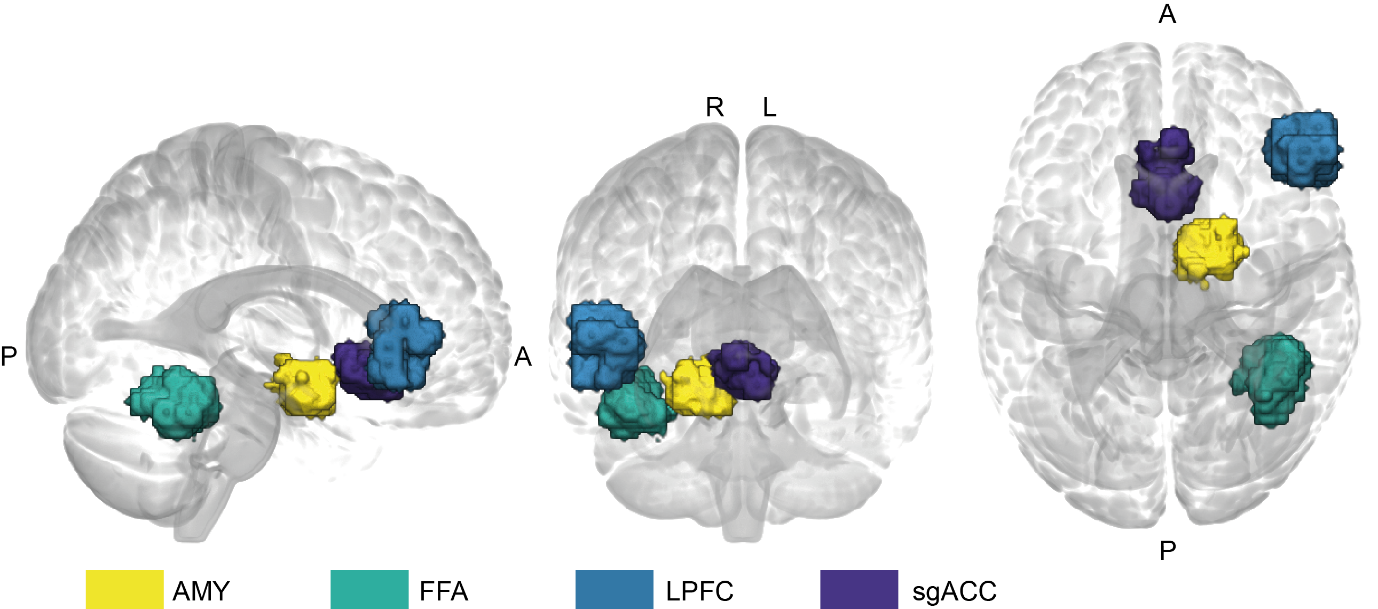


**Figure S8**. Regions of interest selected across individuals for the DCM analysis. A, anterior; P, posterior; L, left; R, right.


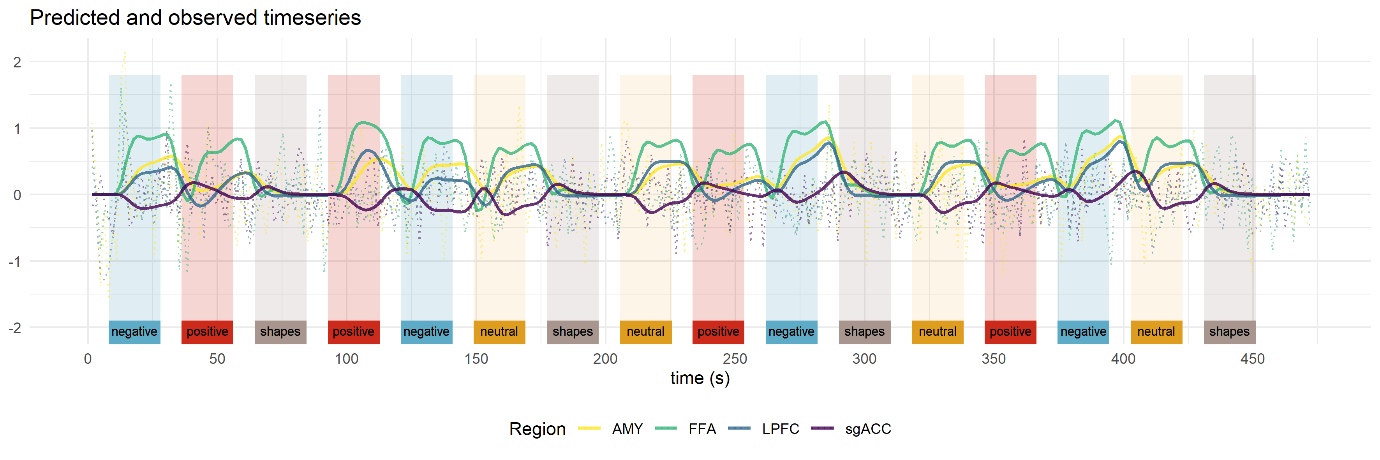


**Figure S9**. Timecourse of a typical subject (dotted) and the prediction (solid) of the DCM for all regions.


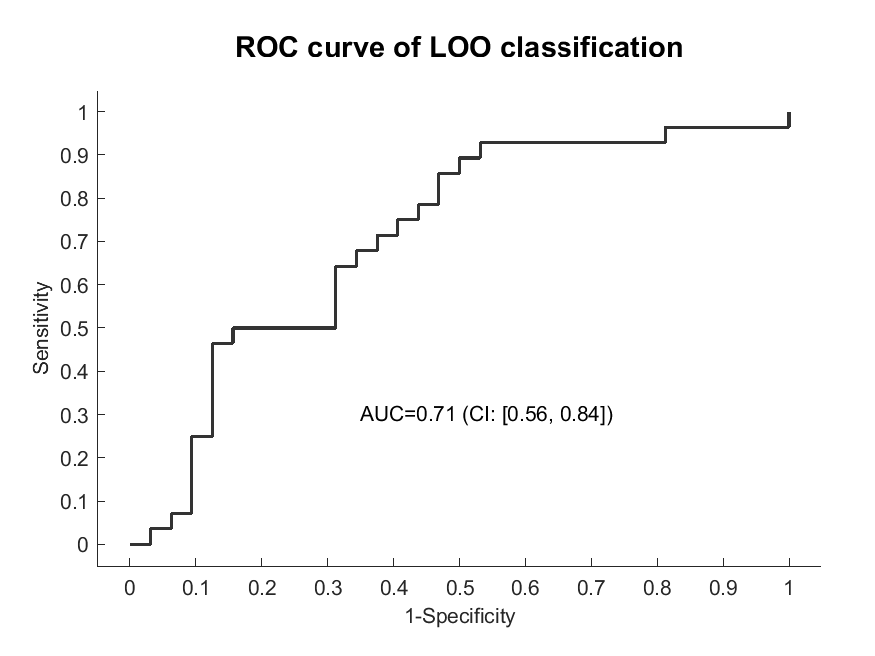


**Figure S10**. Receiver Operation Characteristic (ROC) curve of the leave-one-out cross-validation of the DCM. The area under the curve (AUC) reflects the probability of the model to classify a new participant correctly.

# References

1 Willinger, D., Karipidis, I. I., Beltrani, S., Di Pietro, S. V., Sladky, R., Walitza, S. *et al.* Valence-dependent coupling of prefrontal-amygdala effective connectivity during facial affect processing. *Eneuro* **6** (2019).
